# Design of a chimeric ACE-2/Fc-silent fusion protein with ultrahigh affinity and neutralizing capacity for SARS-CoV-2 variants

**DOI:** 10.1093/abt/tbad001

**Published:** 2023-01-20

**Authors:** Neil M Bodie, Rina Hashimoto, David Connolly, Jennifer Chu, Kazuo Takayama, Bruce D Uhal

**Affiliations:** Paradigm Immunotherapeutics Inc., Monrovia, CA 91016, USA; Center for iPS Cell Research and Application (CiRA), Kyoto University, Kyoto 6068507, Japan; College of Osteopathic Medicine, Department of Medicine, Michigan State University, East Lansing, MI 48824, USA; Innovation Lab, ACROBiosystems, 1 Innovation Way, Newark, DE 19711, USA; Center for iPS Cell Research and Application (CiRA), Kyoto University, Kyoto 6068507, Japan; Department of Physiology, Michigan State University, East Lansing, MI 48824, USA

**Keywords:** lung, ACE-2, coronavirus, therapeutic, fusion protein

## Abstract

**Background:**

As SARS-CoV-2 continues to mutate into Variants of Concern (VOC), there is growing and urgent need to develop effective antivirals to combat COVID-19. Monoclonal antibodies developed earlier are no longer capable of effectively neutralizing currently active VOCs. This report describes the design of variant-agnostic chimeric molecules consisting of an Angiotensin-Converting Enzyme 2 (ACE-2) domain mutated to retain ultrahigh affinity binding to a wide variety of SARS-CoV-2 variants, coupled to an Fc-silent immunoglobulin domain that eliminates antibody-dependent enhancement and extends biological half-life.

**Methods:**

Molecular modeling, Surrogate Viral Neutralization tests (sVNTs) and infection studies of human airway organoid cultures were performed with synthetic chimeras, SARS-CoV-2 spike protein mimics and SARS-CoV-2 Omicron variants B.1.1.214, BA.1, BA.2 and BA.5.

**Results:**

ACE-2 mutations L27, V34 and E90 resulted in ultrahigh affinity binding of the LVE-ACE-2 domain to the widest variety of VOCs, with KDs of 93 pM and 73 pM for binding to the Alpha B1.1.7 and Omicron B.1.1.529 variants, and notably, 78fM, 133fM and 1.81pM affinities to the Omicron BA.2, BA2.75 and BQ.1.1 subvariants, respectively. sVNT assays revealed titers of ≥4.9 ng/ml, for neutralization of recombinant viral proteins corresponding to the Alpha, Delta and Omicron variants. The values above were obtained with LVE-ACE-2/mAB chimeras containing the FcRn-binding Y-T-E sequence which extends biological half-life 3–4-fold.

**Conclusions:**

The ACE-2-mutant/Fc silent fusion proteins described have ultrahigh affinity to a wide variety of SARS-CoV-2 variants including Omicron. It is proposed that these chimeric ACE-2/mABs will constitute variant-agnostic and cost-effective prophylactics against SARS-CoV-2, particularly when administered nasally.

## Teaser

Statement of Significance: A new class of ACE-2/Fc-silent IgG fusion proteins was designed to have ultrahigh affinity and superior neutralizing capacity against a variety of SARS-CoV-2 VOCs. The highest binding affinity (femtomolar) was observed against Omicron variant BA.2. Inclusion of the Y-T-E sequence in the IgG domain is expected to triple biological half-life.

## INTRODUCTION

SARS-CoV-2 has caused the pandemic Coronavirus Disease 2019 (COVID-19), a highly infectious, fatal disease that affects the lungs and other organs. SARS-CoV-2 belongs to the large coronavirus (CoV) family, which are enveloped viruses that have a 26–32 kb, positive-sense, single-stranded RNA genome [1]. The viral envelope consists of a lipid bilayer where the viral membrane (M), envelope (E) and spike (S) structural proteins are anchored. The S protein, also known as viral fusion protein, specifically interacts with its primary receptor, the angiotensin-converting 86 enzyme 2 (ACE-2) on the cell surface to mediate virus-cell fusion, resulting in viral infection through mechanisms thought to be similar to those for SARS-CoV-1 [2]. The viral S-protein binds to its primary receptor ACE-2 through the Receptor Binding Domain (RBD) of the S subunit [3]. A subset of the viral mutations that differentiate variants of SARS-CoV-2 occurs in the RBD portion of the S-subunit [4] and thereby affect binding affinity to the viral receptor. In general, mutations that increase binding affinity of the RBD to ACE-2 result in higher infectivity, but other factors such as immune evasion also play important roles in SARS-CoV-2 virulence [5].

There is an urgent need to develop effective antivirals to combat this newly emerged infectious disease. At the time of this writing, four monoclonal antibodies have been granted Emergency Use Authorization (EUA) by the US FDA for clinical use: (1) bamlanivimab plus etesevimab are neutralizing mAbs that bind to different, but overlapping, epitopes in the spike protein RBD; (2) casirivimab plus imdevimab (REGEN-COV) are recombinant human mAbs that bind to nonoverlapping epitopes of the spike protein RBD; (3) tixagevimab plus cilgavimab (Evusheld) are recombinant human anti-SARS-CoV-2 mAbs that bind nonoverlapping epitopes of the spike protein RBD and (4) sotrovimab targets an epitope in the RBD that is conserved between SARS-CoV and SARS-CoV-2.

Although initially effective at viral neutralization, mAB combinations (1) and (2) have been shown to have significantly reduced neutralizing capacity for the Omicron variant [6, 7]; indeed, on 24 January 2022, the FDA revoked the EUA for REGEN-COV against the Omicron Variant Of Concern (VOC, REGEN-COV Usage Revisions, regencov.com) due to a lack of efficacy against Omicron, and the efficacy of mAB combination (3) for the Omicron variant, initially deemed unclear [8], was recently (23 February 2022) found by the FDA to be reduced to the point of requiring a doubling of the dose of Evusheld used to combat the Omicron VOC. Although sotrovimab was thought to retain significant capacity to neutralize the initial Omicron variants at the time of this writing [9], the FDA subsequently revoked the EUA for sotrovimab on 4 May 2022 (https://www.sotrovimab.com), due to loss of efficacy against Omicron BA.2. Therefore, new variant-agnostic approaches to viral neutralization are needed for the current and future pandemics.

Several research groups have developed mutated ACE-2 mimics, including engineered ACE-2 with optimized binding to the viral RBD [10, 11] and an ACE-2 triple decoy with enhanced affinity for viral variants [12]. Although each of the above engineered ACE-2 mimics had higher affinity binding to the SARS-2 RBD than wild type (w.t.) ACE-2, they all have nanomolar or low, sub-nanomolar binding affinities. In contrast, the data reported herein show binding of our optimized ACE-Fc construct in the picomolar and even femtomolar range to the Omicron BA.1 and BA.2 variants, respectively. In addition, none of the above constructs incorporate the Fc-silent technologies to be discussed below.

In addition to resistance of existing products to newly emerging variants of SARS-CoV-2, the concept of Antibody-Dependent Enhancement (ADE) of viral infection is increasingly being recognized as a serious danger in COVID-19 [13, 14]. In ADE, which was observed in previous outbreaks of dengue virus, Zika virus, Ebola virus, human immunodeficiency virus, Aleutian mink disease parvovirus, Coxsackie B virus and others, pre-existing non-neutralizing or sub-neutralizing antibodies against viral surface proteins, generated during a previous infection, promote the subsequent entry of viruses into the cell during a secondary infection, and thereby intensify the ensuing inflammatory process (12).

However, ADE has not yet been clinically demonstrated in SARS CoV-2 infection. Instead, “Antibody Dependent Inflammation” (ADI) has been documented [15]. Although severe COVID-19 disease is linked to exuberant inflammation, how SARS-CoV-2 triggers inflammation is not well understood. Monocytes and macrophages are sentinel cells that sense invasive infection to form inflammasomes that activate caspase-1 and gasdermin D (GSDMD), leading to inflammatory death (pyroptosis) and release of potent inflammatory mediators. Junqueire *et al*. showed that about 6% of blood monocytes in COVID-19 patients are infected with SARS-CoV-2. Monocyte infection depends on uptake of antibody-opsonized virus by Fcγ receptors, but vaccine recipient plasma does not promote antibody-dependent monocyte infection. SARS-CoV-2 begins to replicate in monocytes, but infection is aborted, and infectious virus is not detected in infected monocyte culture supernatants. Instead, infected cells undergo inflammatory cell death (pyroptosis) mediated by activation of NLRP3 and AIM2 inflammasomes, caspase-1 and GSDMD. Moreover, tissue-resident macrophages, but not infected epithelial and endothelial cells from COVID-19 lung autopsies, have activated inflammasomes. These findings, taken together, suggest that antibody-mediated SARS-CoV-2 uptake by monocytes/macrophages triggers inflammatory cell death that aborts production of infectious virus, but causes systemic Fc receptor-dependent inflammation that contributes to COVID-19 pathogenesis [15].

For all the reasons above, the objective of this work was to design molecules with high but variant-agnostic binding affinities to a wide variety of SARS-CoV-2 variants, particularly if they might also incorporate features that reduce or eliminate ADI or ADE. In this report, we describe the design of just such molecules, which combine a synthetic human ACE-2 domain containing mutations that allow variant-agnostic, ultra-high affinity binding to the SARS-CoV-2 S1 subunit, combined together with an Fc-silent antibody domain that essentially eliminates the potential for ADI or ADE. Moreover, a third mutant option of the antibody domain of the chimera is offered, with the intent to substantially increase (3–4-fold) the biological half-life of the chimera if delivered by aerosol or nasal administration. Given that nasal infection is the most likely route of SARS-CoV-2 entry in humans [16], it is proposed that a nasal administration of the new chimeric molecules described herein will constitute an effective prophylactic against SARS-CoV-2 infection that will not only be effective, but also is expected to be economically far superior to current monoclonal antibody treatments for COVID-19.

## MATERIALS AND METHODS

### Viral and protein constructs

Viral RBD, S1 protein subunit and S1 subunit trimers were synthesized by Absolute Antibody (Cleveland, United Kingdom) as recombinant proteins designed on the basis of publicly available sequence data (outbreak.info). Recombinant human ACE-2 (rhACE-2) constructs were also synthesized by Absolute Antibody (Boston, MA) designed on the basis of sequence data obtained from NCBI protein sequence data and modified as described in (Figs 2–5). GenScript IgG FL18–740 (Cat. No. Z03516) was purchased from GenScript (Piscataway NJ). The ACE-2/mAB chimeras were synthesized by Absolute Antibody (Boston, MA).

### In silico molecular modeling

Molecular models were derived from publicly available ACE-2 and SARS-CoV-2 sequence databases (Outbreak.info) with Protean 3D Version 17.3 (DNASTAR. Madison, WI) software using the method described by Zhang and Zhang [17], which uses a knowledge-based potential to solve protein folding and protein structure prediction problems. Based on the parent software I-TASSER [18], the method of Zhang and Zhang can differentiate well between Leucine and Isoleucine, an ability important for the potential analysis of Leucines in viral or ACE-2 variants. Comparisons of atomic-level structures and viral-ACE-2 interactions were achieved with the knowledge-based atomic potential algorithm DFIRE [19], which generates a numerical protein–protein interaction score that becomes more negative with more stabilizing molecular interactions. Mutations in ACE-2 yielding the highest affinity binding to the widest variety of SARS-2 variants were sought. To find these, dozens of in silico experiments were performed to determine the optimal ACE2 mutations (L,V) for the most highly conserved SARSCoV2 RBD anchor amino acid (AA) residues RBD L455, F456 and Y473, based on DMS of the SARSCoV2 RBD, see (Fig. 3B) [20]. The final mutation we chose, ACE2 N90E, was based on the DNASTAR modeling program suggesting steric hindrance of the ACE2 N90 glycan.

### Binding affinity determinations

Surface Plasmon Resonance (SPR) assays of protein–protein interactions were performed by Acro Biosystems (Beijing Economic Development Zone, Beijing China) on a Biacore T200 Instrument fitted with Series SCM5 Sensor Chip. Prior to SPR assay, samples were desalted on Zeba Spin 7 K MWCO columns. Binding affinities were determined in HBS-N buffer, 10X (0.1 M HEPES, 1.5 MNaCl) containing EDTA and Tween 20, at a flow rate of 30 μL/min, run for 120 s association and 180 s dissociation. The reference subtracted SPR binding curves were blank subtracted, and curve fitting was performed with a 1:1 model to obtain kinetic parameters using the Biacore T200 Evaluation software. Binding data are reported as estimated dissociation constant (KD, Figs 6 and 10 and Table 1).

**Table 1:** Binding affinities of paradigm’s ACE-2/fusion protein chimeras to SARS-CoV-2 variant constructs

ACE-2/IgG Chimera	SARS-CoV-2 Construct	K_D_ by SPR^*^
LiVE/STR chimera	Alpha variant B.1.1.7, S1 subunit	378 pM
LiVE-Longer YTE chimera	Alpha variant B.1.1.7, S1 subunit	93 pM
LiVE/STR chimera	Delta variant B.1.617.2, RBD only	554 pM
LiVE-Longer YTE chimera	Delta B.1.617.2, RBD only	507 pM
LiVE/STR chimera	Omicron BA.1 spike protein trimer	144 pM
LiVE-Longer YTE chimera	Omicron BA.1 spike protein trimer	73 pM
LiVE-Longer YTE chimera	Omicron BA.2 spike protein trimer	78 fM
LiVE-Longer YTE chimera	Omicron BA2.75 spike protein trimer	133 fM
LiVE-Longer YTE chimera	Omicron BA.5 spike protein trimer	5.43 pM
LiVE/STR chimera	Omicron B.1.1.529^*^^*^ RBD only	308 pM
LiVE-Longer YTE chimera	Omicron B.1.1.529^*^^*^ RBD only	402 pM
LiVE-Longer YTE chimera	Omicron BA4.6 spike protein trimer	845 pM
hACE-2, Fc tag (ACRO)^*^^*^^*^	Omicron BA4.6 spike protein trimer	27.1 nM
LiVE-Longer YTE chimera	Omicron BQ.1.1 spike protein trimer	1.81 pM
hACE-2, Fc tag (ACRO)^*^^*^^*^	Omicron BQ.1.1 spike protein trimer	12.6 nM
LiVE-Longer YTE chimera	Omicron XBB.1 spike protein trimer	215 pM
hACE-2, Fc tag (ACRO)^*^^*^^*^	Omicron XBB.1 spike protein trimer	22.4 nM
hACE-2, Fc tag (ACRO)^*^^*^^*^	Wuhan variant, S1 subunit	16.0 nM
hACE-2, Fc (Genscript) ^*^^*^^*^^*^	Wuhan variant, S1 subunit	3.0 nM

^*^SPR assay (Acro Biosystems)

^**^Omicron variant B.1.1.529, 01 November 2022

^***^Human ACE2, Fc Tag (Cat. No. AC2-H5257, ACRO Biosystems)

^****^Human ACE-2 Fc fusion protein (Cat. No. Z03516, Genscript)

### Surrogate viral neutralization tests

The surrogate Viral Neutralization Test (sVNT, C-PASS, GenScript USA Inc., Piscataway, NJ 08854) was used to characterize the antibodies described, using the methodology defined previously [21]. The manufacturer’s instructions were followed without modification except where specifically noted below. Briefly, the SARS-CoV-2 sVNT Kit is a blocking ELISA detection tool, which mimics the virus neutralization process. The kit contains two key components: the Horseradish peroxidase (HRP) conjugated recombinant SARS-CoV-2 RBD fragment (HRP-RBD) and the human ACE2 receptor protein (hACE2). The protein–protein interaction between HRP-RBD and hACE2 can be blocked by neutralizing antibodies against SARS-CoV-2 RBD; specific applications are described in the Legends to Figures.

### SARS-CoV-2 preparation

The SARS-CoV-2 strains B.1.1.214 (GISAID accession number: EPI_ISL_2897162), BA.1 (GISAID accession number: EPI_ISL_9638489), BA.2 (GISAID accession number: EPI_ISL_11900505) and BA.5 (GISAID accession number: EPI_ISL_14018094) were isolated from a nasopharyngeal swab sample of a COVID-19 patient. This study has been approved by the research ethics committee of Kyoto University. The virus was plaque-purified and propagated in TMPRSS2/Vero cells (JCRB1818, JCRB Cell Bank) [22]. SARS-CoV-2 was stored at −80°C. All experiments including virus infections were done in biosafety level 3 facilities at Kyoto University strictly following regulations.

### Airway organoids preparation

Airway organoids (AOs) were generated according to our previous report [23]. Briefly, normal human bronchial epithelial cells (NHBE, Cat. No. CC-2540, Lonza) were used to generate AO. NHBE were suspended in 10 mg/ml cold Matrigel growth factor reduced basement membrane matrix (Corning). About 50 μL of cell suspension was solidified on pre-warmed cell-culture-treated multi-dishes (24-well plates; Thermo Fisher Scientific) at 37°C for 10 min, and then 500 μL of expansion medium was added to each well. AO were cultured with AO expansion medium for 10 days. To mature the AO, expanded AOs were cultured with AO differentiation medium for 5 days. In experiments evaluating the antibodies, AOs were dissociated into single cells, and then were seeded into 96-well plates.

### Determination of antiviral effects

Human AOs prepared as described above were challenged at 24 h of culture with virus (MOI = 0.1) in the presence of absence of fusion proteins applied at 0.0064, 0.032, 0.16, 0.8, 4, 20 and 100 μg/ml (*n* = 3). Twenty four hours later, media were replaced (with fusion proteins). Twenty four hours thereafter, supernatants were harvested and prepared for determination of viral copy number as described below.

### Quantification of viral RNA copy number

The cell culture supernatant was mixed with an equal volume of 2 × RNA lysis buffer (distilled water containing 0.4 U/μL SUPERase ITM RNase Inhibitor (Thermo Fisher Scientific), 2% Triton X-100, 50 mM KCl, 100 mM Tris–HCl (pH 7.4) and 40% glycerol) and incubated at room temperature for 10 min. The mixture was diluted 10 times with distilled water. Viral RNA was quantified using a One Step TB Green PrimeScript PLUS RT-PCR kit (Perfect Real Time) (Takara Bio) on a StepOnePlus real-time PCR system (Thermo Fisher Scientific). The primers used in this experiment are as follows: (forward) AGCCTCTTCTCGTTCCTCATCAC and (reverse) CCGCCATTGCCAGCCATTC. Standard curves were prepared using SARS-CoV-2 RNA (10^5^ copies/μL) purchased from Nihon Gene Research Laboratories.

### Determination of ACE-2 enzyme activity

The enzyme activity of ACE-2 within the chimeric fusion proteins was determined with a commercially available ACE-2 assay kit (BPS Bioscience, San Diego, CA). The manufacturer’s instructions were followed directly, and the enzyme activity was expressed as fluorescence units of fluorogenic substrate converted in 30 min by equal amounts of either purified rhACE-2 or fusion protein.

## RESULTS

**Figure 1:**
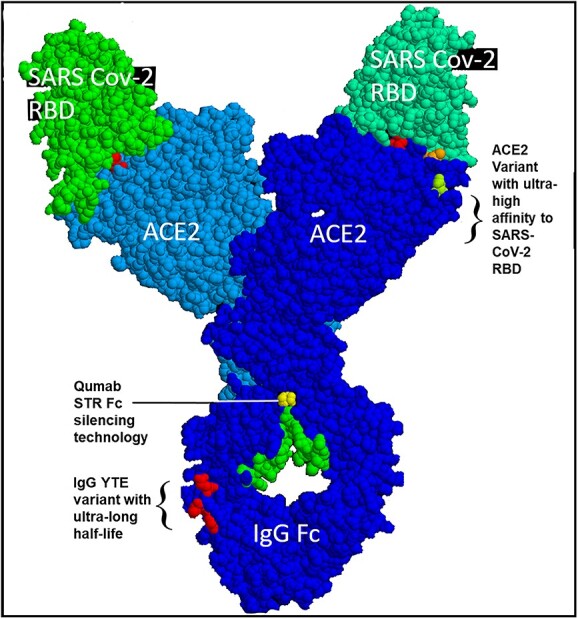
Critical features of Paradigm’s chimeric ACE-2/Fc-silent antibody technology. These include: the use of a full length human ACE-2 domain (AAs 18–740, blue), which when modified as described next, substantially increases binding affinity for SARS CoV-2 RBD/spike protein (green); the choice of specific mutations in ACE-2 (top right red orange, light green AAs) that impart picomolar affinity for the RBD and ~ femtomolar affinity for the full-length spike protein; linkage of the ACE-2 construct to a completely Fc-silent “STR” mutated monoclonal antibody (yellow, Mike Clark PhD, used with permission). The use of STR technology in an ACE2 chimeric prophylactic for SARS CoV-2 is patent pending, see (Fig. 15). In addition, one of the two mAb chimeras (“LiVE-Longer” vs. “LiVE”) utilizes a Y-T-E variant (red AAs, bottom) for increased half-life by binding to FcRn, which recycles IgG and is thereby predicted to increase its biological half-life by 3–4-fold (see Fig. 16 and text for details). The “LiVE” mAB does not contain the YTE sequence; both LiVE and LiVE-Longer are tested in (Figs 11–13 and Table 1). This figure was generated by Protean 3D, Version 17.3 (DNASTAR. Madison, WI).

**Figure 2:**
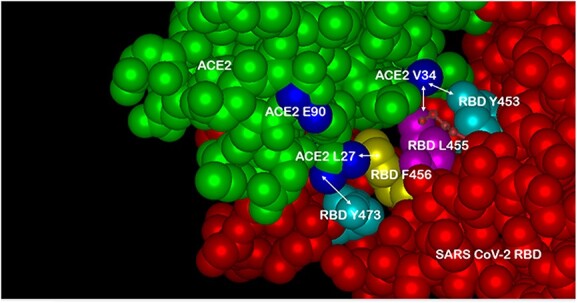
Three-dimensional model of the SARS-CoV-2/ACE-2 molecular interface. Multiple In Silico modeling programs were used to predict optimal AA substitutions in ACE-2 (green) that would retain high binding affinity to the widest possible range of AA variations in the SARS-CoV-2 spike protein RBD (RBD, red). Two of the three optimal substitutions in ACE-2 depicted here are: T27L (ACE2 L27, blue lower) and H34V (ACE2 V34, blue upper right), shown here in close proximity to RBD AAs Y473 and F456 versus L455 and Y453, respectively. The third substitution N90E (ACE2 E90, blue upper left) is discussed in (Fig. 3). See text for details.

**Figure 3:**
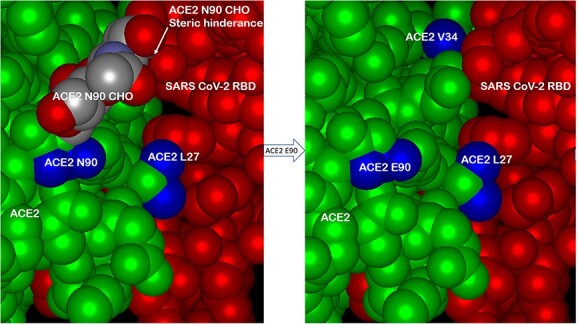
The effect of eliminating the glycosylation site at AA 90 of ACE-2. The asparagine at AA 90 of w.t. ACE-2 (N90) is a site for N-linked glycosylation (left panel, ACE-2 N90 CHO) which, when the sugar is present, causes steric hindrance of ACE-2/RBD interaction. Multiple modeling platforms predicted that elimination of the glycosylation site by the mutation N90E (asparagine to glutamate) would relieve steric hindrance and allow closer ACE-2/RBD interaction, particularly in combination with the ACE-2 H34V substitution (right panel, top). Subsequent figures below show data consistent with this prediction. See text for details.

**Figure 4:**
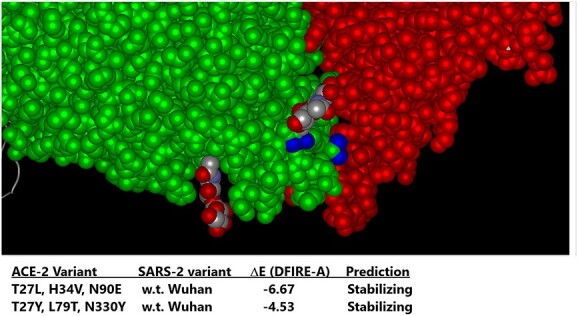
The designed ACE-2 variant LVE/STR chimera scores higher than other ACE-2 Fusion protein competitors by the DFIRE scoring method. Molecular modeling was conducted with Protean 3D Version 17.3 (DNASTAR. Madison, WI) software, aimed at testing Paradigms’ proprietary LiVE variant (ACE-2 mutations T27L, H34V, and N90E) binding to w.t. SARS-2 RBD (DFIRE = −6.67), as it compares to the ACE-2 variant YTY (ACE-2 mutations T27Y, L79T and N330Y) binding to the w.t. SARS-2 RBD (DFIRE = −4.53). A lower DFIRE score predicts tighter (stabilizing) protein–protein interactions. See Methods for details.

**Figure 5:**
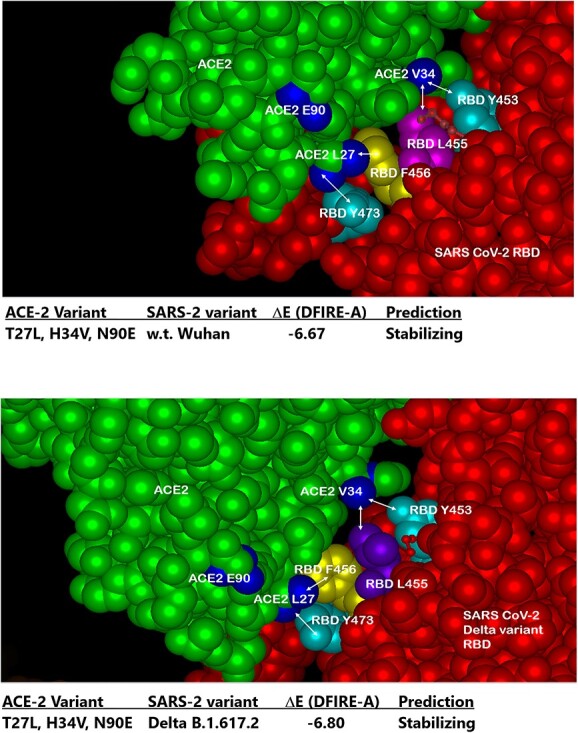
In silico testing of SARS CoV-2 Delta variant predicts Paradigm’s ACE-2 variant LVE/STR chimera out-scores competing ACE-2 chimeric technologies. Top panel: molecular modeling with Protean 3D Version 17.3 (DNASTAR. Madison, WI) software of ACE-2 LVE construct (green) binding to w.t. SARS-CoV-2 RBD (red) yields DFIRE score = −6.67. Bottom panel: identical modeling of ACE-2 LiVE construct (green) binding to SARS-CoV-2 Delta variant yields DFIRE score = −6.80, slightly tighter (stabilizing) than that for the w.t. RBD. Note rotation of RBD F456 (yellow) in Delta variant toward ACE-2 AA 27, made permissive by the T27L mutation.

**Figure 6:**
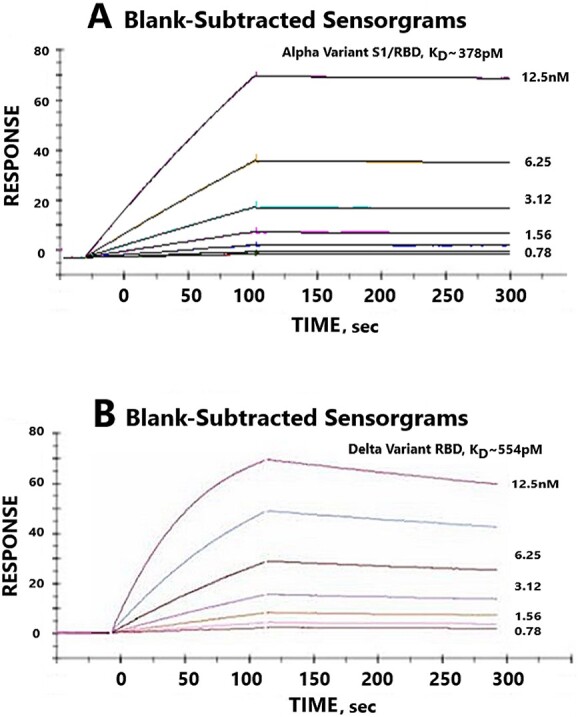
High binding affinity of Paradigm’s ACE-2 variant LVE/STR chimera to the RBD of Alpha and Delta variants of SARS-CoV-2. SPR assays were performed to determine binding affinities between the synthesized, purified LVE/STR chimera and synthesized RBDs of SARS-CoV-2 Alpha or Delta variants. Panel A: the S1 subunit with RBD of SARS-CoV-2 Alpha variant B.1.1.7 was synthesized, purified and subjected to SPR assay against the purified LVE/STR chimera. Determined binding affinity was 0.378 nM (Acro Biosystems). Note slow “off rate” compared with Panel B. Panel B: the RBD of Delta variant B.1.617.2 was synthesized, purified and subjected to SPR assay against the purified LiVE/STR chimera. Determined binding affinity was 0.554 nM (Acro Biosystems). See (Table 1) for more complete listing of binding affinities.

The basic design of Paradigm’s ACE-2-Fusion protein chimera is shown in (Fig. 1). A synthetic, mutant variant of human ACE-2 was designed to have high-affinity binding to a wide variety of SARS-CoV-2 variants including Delta and Omicron (data shown below). The ACE-2 was linked to an Fc-silent designer monoclonal antibody of two forms: Fc-silent (L234S/L235T/G236R, “STR” [26]) and a “live longer” Fc-silent version containing the YTE variant (M252Y/S254T/T256E [27]), which increases binding to FcRn receptor to achieve longer biological half-life as discussed below. (Fig. 2) shows a three-dimensional model of the ACE-2/SARS-CoV2 molecular interface with the ACE-2 mutations found to impart high binding affinity to the widest range of SARS-2 variants. In particular, the AA substitutions T27L and H34V interact with SARS-2 RBD AAs 473 and 456 versus 455 and 453, respectively. The third substitution N90E (ACE2 E90) is discussed next.

 (Fig. 3) shows the effect of the ACE-2 AA substitution N90E, which eliminates the site for N-linked glycosylation of ACE-2 at the ACE-2/SARS-2 interface. Surprisingly, elimination of the N-linked glycan resulted in higher affinity binding to several SARS-2 variants, presumably due to loss of steric hindrance otherwise caused by the glycan. In (Fig. 4), molecular modeling with Protean 3D software of ACE-2 variant binding to a purified, recombinant w.t. SARS-2 RBD revealed tighter binding of the ACE-2 LVE variant (DFIRE score − 6.67), than that of the ACE-2 AA substitutions YTY at positions 27, 79 and 330, respectively (DFIRE score − 4.53). See Methods for details.

 (Fig. 5) shows three dimensional molecular models and binding affinity predictions for the interactions between Paradigm’s ACE-2 LVE/STR chimeric Fusion protein and the purified recombinant RBDs of the w.t. SARS-2 variant (top panel) versus the RBD of the Delta B.1.617.2 variant (bottom panel). The lower DFIRE score of −6.80 for binding to the Delta variant predicts a more stable (tighter) ACE-2/SARS-2 interaction. Consistent with this prediction, the data in (Fig. 6) show SPR data indicating very high binding affinity of Paradigm’s ACE-2 LVE/STR chimera to either the purified S1/RBD of the Alpha B.1.1.7 variant (378 pM) or the purified RBD of the Delta B.1.617.2 variant (554 pM).

**Figure 7:**
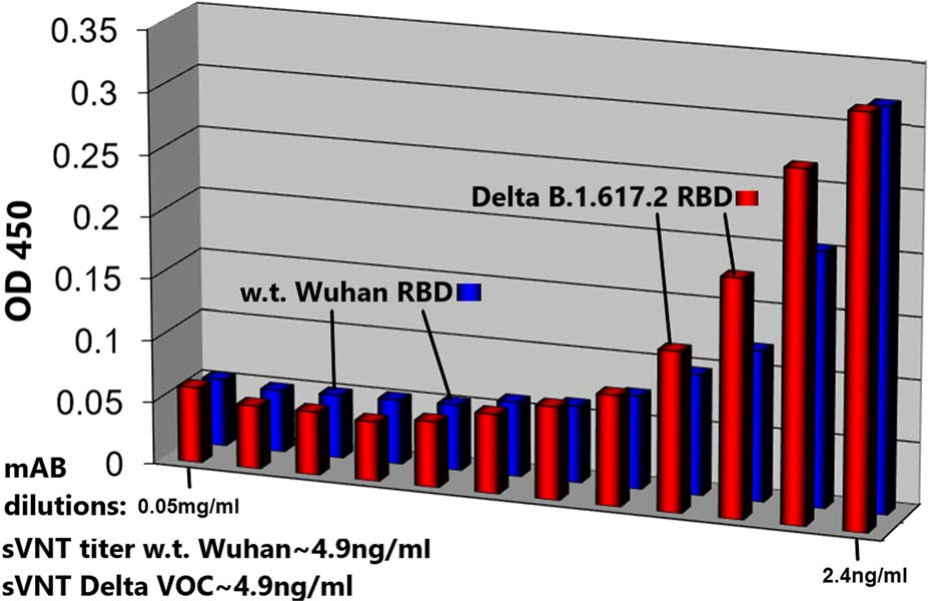
Paradigm’s ACE-2 variant LVE/STR chimera neutralizes Delta Variant B.1.617.2 RBD or w.t. SARS-CoV-2 RBD (Wuhan strain) with nearly equal potency. sVNTs (see Methods) were performed with SARS-CoV-2 RBD proteins expressing the Delta Variant B.1.617.2 sequence (T478K and L452R mutants, red bars) or the w.t. (original Wuhan strain) sequence (blue bars) incubated with the ACE-2 LiVE/STR construct. Dilutions shown are 0.05–2.4 ng/ml, left to right. Note similar sVNT titers of ~ 4.9 ng/ml.

**Figure 8:**
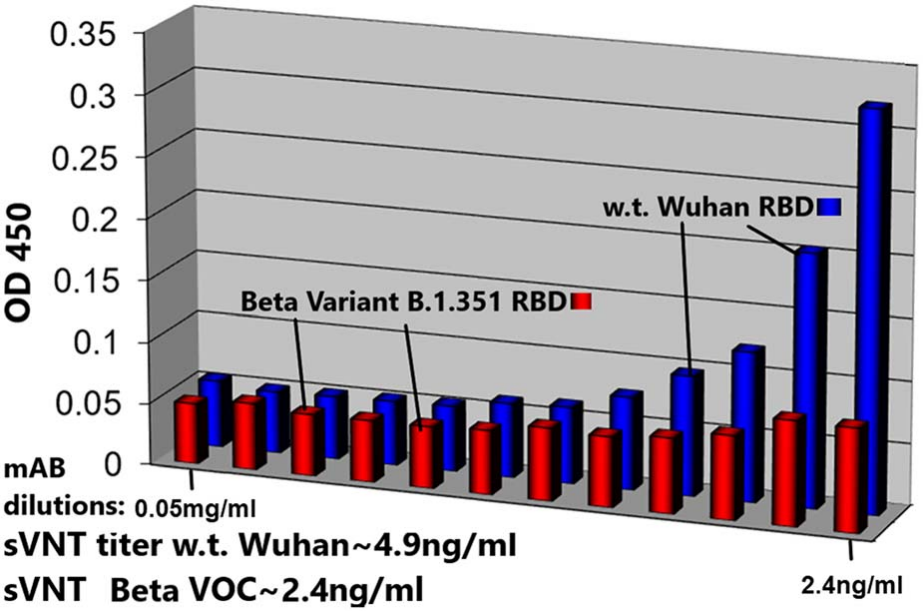
Paradigm’s ACE-2 variant LVE/STR chimera neutralizes Beta Variant B.1.351 RBD significantly better than w.t. SARS-CoV-2 RBD. sVNT assays were performed with SARS-CoV-2 RBD proteins expressing the Beta Variant B.1.351 sequence (K417N, E484K and N501Y mutants, red bars) or the w.t. (original Wuhan strain) sequence (blue bars) incubated with the ACE-2 LiVE/STR construct. Dilutions shown are 0.05–2.4 ng/ml, left to right. Note sVNT titers of ~ 2.4 ng/ml for neutralization of the SARS-2 Beta variant.

**Figure 9:**
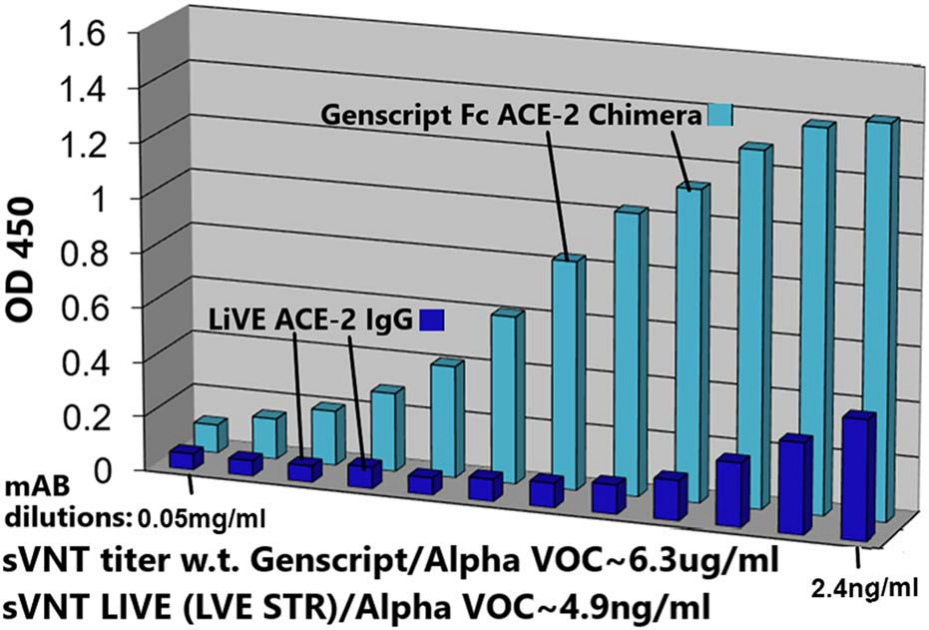
Paradigm’s ACE-2 variant LVE/STR chimera neutralizes Alpha Variant B.1.1.7 RBD significantly better than GenScript IgG FL18–740 w.t. ACE-2 mAB. sVNT assays were performed with SARS-CoV-2 RBD proteins expressing the Alpha Variant B.1.1.7 sequence (N501Y mutant) challenged with either Paradigm’s ACE-2 variant LVE/STR chimera (dark blue bars) or with the GenScript IgG FL18–740 w.t. ACE-2 mAB (light blue bars, binding affinity 3 nM). Dilutions shown are 0.05–2.4 ng/ml, left to right. Note sVNT titers of ~ 6.3 μg/ml for the Genscript mAB for neutralization of the Alpha variant, in contrast to ~ 4.9 ng/ml for neutralization of the Alpha variant by the Paradigm construct LiVE-STR.

**Figure 10:**
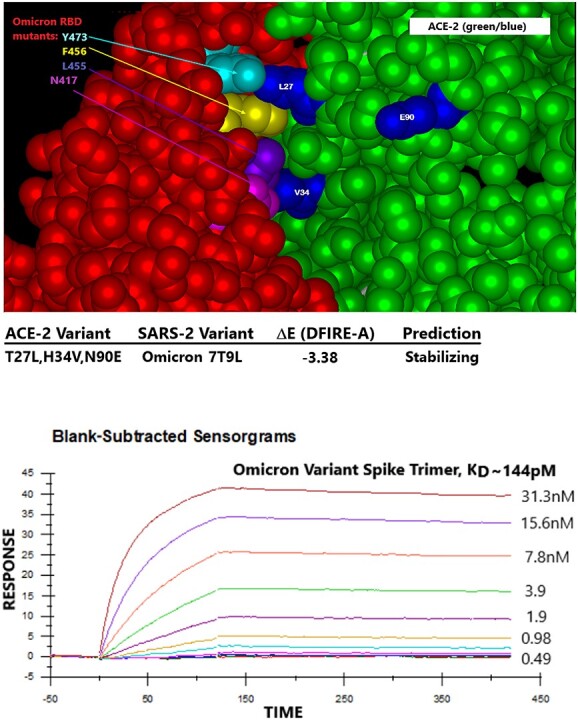
Paradigms’ ACE-2 variant LVE/STR chimera binds to SARS-2 Omicron variant B.1.1.529 spike protein trimer with high affinity. Top panel: molecular modeling of Paradigms’ ACE-2 variant LVE/STR chimera binding to the Omicron variant sequence as of 1 November 2022. Omicron RBD (red) mutations Y473, F456, L455 and N417 are shown at upper left, and ACE-2 (green) mutations LVE are shown in blue. The ACE-2 H34V makes contact with the aliphatic straight chain of Omicron mutation Q493R and K417N. Note the vertical orientation of N417 in close contact with V34, which enabled higher affinity binding compared to the w.t. K417, which assumed a more horizontal orientation in earlier SARS-2 variants. Molecular modeling performed identically to that in (Figs 4 and 5) yields superior DFIRE score of −3.38 (stabilizing). See Discussion for details. Bottom panel: the S1 subunit trimer of SARS-CoV-Omicron variant described above was synthesized, purified and subjected to SPR assay against the purified LVE/STR chimera. Determined binding affinity was 0.144 nM (Acro Biosystems); see (Table 1) for more complete listing of binding affinities.

**Figure 11:**
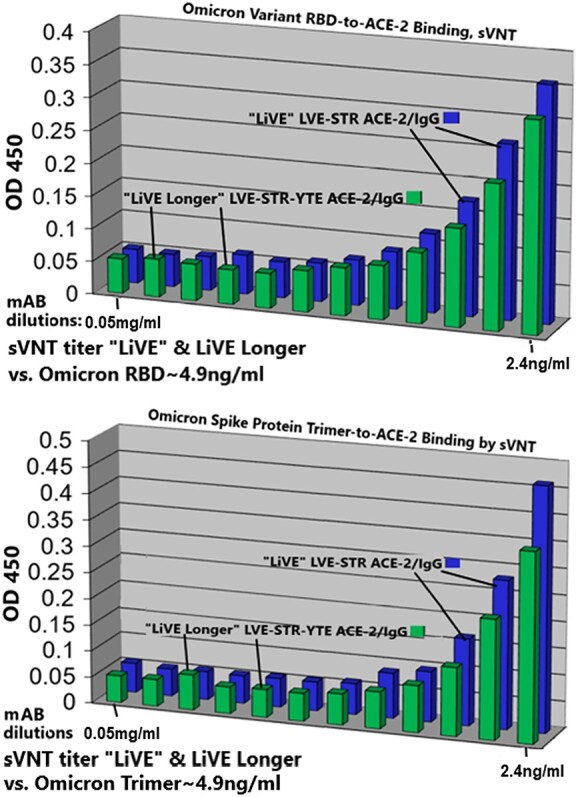
Paradigm’s ACE-2 variant LVE/STR chimeras potently neutralize the SARS-CoV-2 Omicron Variant B.1.1.529. sVNT assays were performed with purified recombinant SARS-CoV-2 RBD (top panel) or spike protein trimers (bottom panel) expressing the Omicron variant sequences described in (Fig. 10). The bargraphs show inhibition of the binding of Omicron RBD (top) or Omicron spike protein trimer (bottom) to purified recombinant ACE-2 by either Paradigm’s “LiVE” ACE-2 variant LVE/STR STR Fusion protein chimera or by the “LiVE Longer” LVE/STR STR–YTE IgG chimera. Dilutions shown are 0.05–2.4 ng/ml, left to right. Note similar sVNT titers of ~ 4.9 ng/ml for neutralization of Omicron RBD or Omicron spike trimers, and slightly better neutralization by the “LiVE Longer” chimera (green). See (Figs 1 and 13) for additional details about the ACE-2/Fusion protein chimeras.

sVNTs are also consistent with the above results; (Fig. 7) displays sVNT data indicating that Paradigm’s ACE-2 LVE/STR chimeric Fusion protein could neutralize the purified recombinant RBD of either the w.t. SARS-2 Wuhan variant (blue bars) or the Delta variant B1.617.2 (red bars) with nearly equal potency (sVNT titer ~ 4.9 ng/ml). In (Fig. 8), the LVE/STR chimera neutralized the recombinant RBD of the Beta variant B.1.351 with greater potency (red bars, sVNT titer ~ 2.4 ng/ml) than that for the RBD w.t. Wuhan variant (blue bars, titer ~ 4.9 ng/ml). In sVNT tests of the SARS-2 Alpha variant (Fig. 9), the LVE ACE-2/STR chimera neutralized the purified RBD of the Alpha variant B1.1.7 significantly better (sVNT titer > 4.9 ng/ml) than the Genscript Fc-IgG/ACE-2 chimera Z03516 (~6.3 μg/ml).

 (Fig. 10) displays molecular modeling of the molecular interface between Paradigms’ ACE-2 variant LVE/STR chimera and the RBD of the Omicron variant of SARS-CoV-2 (RBD sequence as of 12 October 2021 at 75% cutoff). Of note, the aliphatic side chain of the Omicron Q493R mutation (purple) makes contact with ACE-2 mutation V34. In molecular modeling (see Methods), simulation of the ACE-2 LVE/STR chimera binding to the Omicron variant yielded a very favorable DFIRE score of −7.26, indicating a tight, stabilizing interaction. This modeling is consistent with the sVNT results in (Fig. 11), which show that the Omicron variant sequence known on 9 December 2022, when expressed as either a purified recombinant RBD (top panel) or purified spike protein trimer (bottom panel), could be potently neutralized by Paradigm’s “LiVE” ACE-2 variant LVE/STR STR Fusion protein chimera or by the “LiVE Longer” LVE/STR STR—YTE Fusion protein chimera (sVNT titers ~4.9 ng/ml).

**Figure 12:**
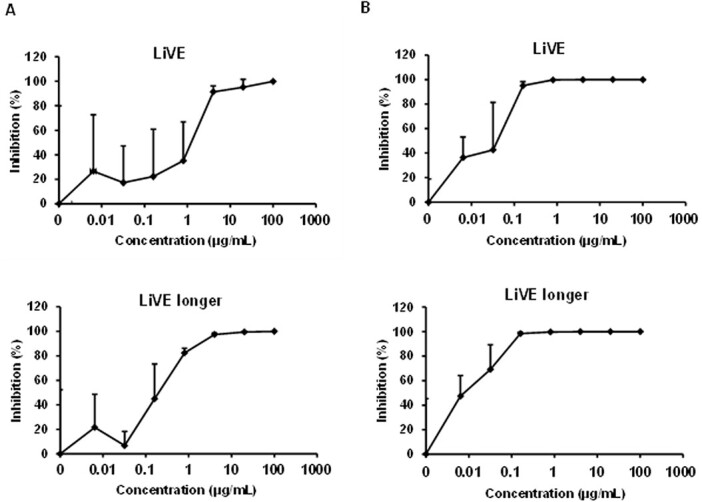
Antiviral effects of “LiVE” or “LiVE Longer” fusion proteins against SARS-CoV-2 B.1.1.214 or BA.1 infection of human AO cultures. After 1 day in culture, organoid cultures (1.0 × 10^4^ cells/well) were infected with 0.1 MOI SARS-CoV-2 B.1.1.214 (A) or BA.1 (B) and then cultured with the medium containing a serially diluted antibody for 2 days. The viral RNA copy number in the cell culture supernatant was measured by qPCR. Data are represented as means ± SD (*n* = 3). See Methods for details.

**Figure 13:**
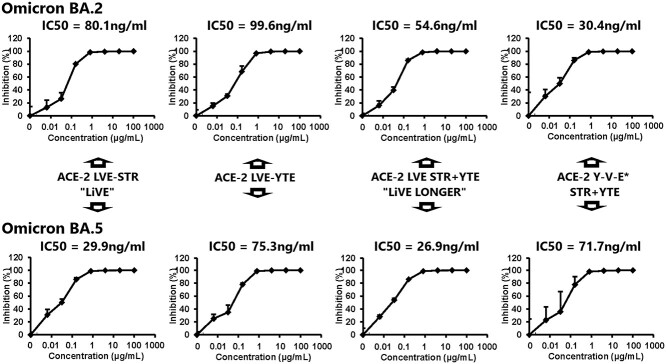
Antiviral effects of the “LiVE”, “LiVE Longer” or related chimeric fusion proteins against SARS-CoV-2 Omicron BA.2 or BA.5 infection of human AO cultures. Organoid cultures were exposed to Omicron variants BA.2 (top panels) or BA.5 (bottom panels) as described in Materials and Methods and were challenged with the fusion proteins “LiVE” (leftmost pair), “LiVE Longer” (3rd pair from left), modified LiVE without the STR motif (2nd from left) or a modified LiVE LONGER with tyrosine substituted for leucine at position 27 (Y-V-E^*^, rightmost panels). Note the most potent inhibition of Omicron BA.5 replication by the LiVE (IC50 = 29.9 ng/ml) and LiVE LONGER fusion proteins (IC50 = 26.9 ng/ml). See text for details.

In (Fig. 12), The LiVE and LiVE Longer fusion proteins were highly effective at inhibiting viral replication of the B.1.1.214 (left) or BA.1 (right) SARS-CoV-2 variants when applied for 2 days to human AO cultures exposed to virus [23]. Half-maximal inhibition of viral replication was obtained by the “LiVE Longer” fusion protein at 202 and 9.3 ng/ml for the B.1.1.214 and BA.1 variants, respectively. (Fig. 13) displays the antiviral effects of the LiVE or LiVE Longer chimeras and related constructs against the Omicron variants BA.2 and BA.5. Although all constructs potently inhibited viral replication, the most potent inhibition was observed for the LiVE and LiVE Longer chimeras against Omicron BA.5, with IC50s of 29.9 and 26.9 ng/ml, respectively.

**Figure 14:**
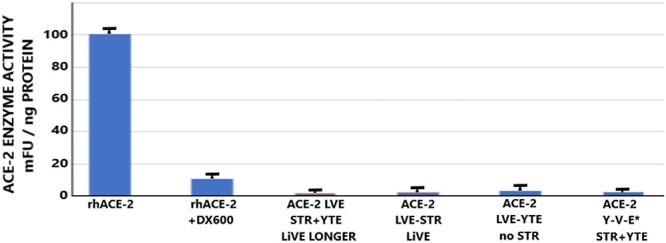
ACE-2 enzymatic activity of the chimeric fusion proteins. Equal amounts of rhACE-2 were subjected to measurements of ACE-2 enzyme activity as described in Materials and Methods, using standard ACE-2 enzyme assay methods based on fluorogenic substrate conversion. Results are the mean ± S.E.M. of eight replicates per group. Note inhibition of ACE-2 activity by the competitive inhibitor peptide DX600 (at 1 μM) and the extremely low to negligible activity in any of the chimeric fusion proteins tested. See text for details.

**Figure 15:**
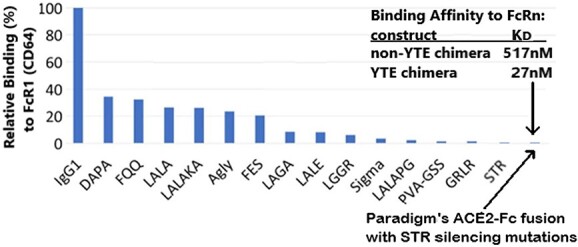
Paradigm's Fc fusion protein including the STR Fc silencing mutations licensed by MAbsolve is Superior to LALA and other Reduced Fc Effector Function Technologies. A comparison of Fc-silencing technologies shows the relative binding of purified IgG1 (control) versus a variety of Fc-mutant antibody technologies to immobilized human FcγRI (CD64), as determined by SPR assay (data courtesy of MAbsolve, https://mabsolve.com/science/#linkone). Note extremely low binding of either the YTE variant used by Paradigm (far right bar) or MAbsolve’s STR variant. Inset: The binding affinities of the non-YTE LiVE or YTE-variant LiVE-Longer ACE-2/Fusion protein chimeras to purified FcRn receptor [24] were determined by SPR assay (Acro Biosystems). Note high affinity binding of the YTE chimera (27 nM) to FcRn. See text for details.

**Figure 16:**
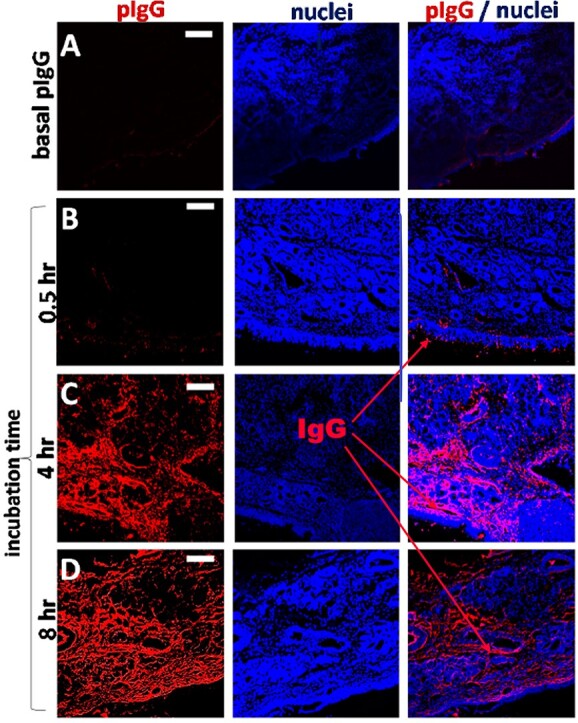
Longer half-life of nasally applied chimeric ACE-2/Fc-silent Fusion protein is due to kinetics of FcRn-Dependent IgG Uptake into Olfactory Mucosa. Nasally applied IgG is slowly transcytosed through binding to FcRn receptor expressed in porcine or human olfactory epithelia. In this figure, Ladel *et al*. [24] used ex vivo porcine olfactory mucosa to show to track the uptake of allogenic IgG (red) into porcine olfactory epithelia. (A) Basal levels of endogenous pIgG (porcine IgG) were detected with a low signal at the apical side, in the basal cell layer, glands, cavernous bodies and blood vessels. This signal served as a blank and was subtracted from the photos showing the penetration of exogenous pIgG. (B) After 30 min, only the areas close to the apical side show immunoreactivity for pIgG, but some signal was detected in the lamina propria. (C) After 4 h, the pIgGs obviously distributed into the lamina propria. ^*^Indicate round structure filled with cells and mostly spared from IgG (D) After 8 h, pIgG were detected throughout the whole lamina propria. Nuclei stained with DAPI; epithelial control: quality control for tissue integrity, stained with HE. Scale bar: 100 μm. On this basis, the YTE variant of Paradigm’s ACE-2 LVE/IgG YTE chimera (which binds longer to FcRn (see Zhu *et al.* [25]) is predicted to have 3–4-fold longer biological half-life when administered nasally. Reprinted with publisher’s permission from Ladel *et al*., Allogenic Fc Domain-Facilitated Uptake of IgG in Nasal Lamina Propria. Pharmaceutics, 10:107, 2018. See text for details.

Data in (Fig. 14) revealed that the chimeric fusion proteins retained very little to no enzymatic activity, when compared with equal amounts of rhACE-2. Although the reasons for the lack of enzyme activity are not presently clear, they may include steric hindrance caused by the fusion of the ACE-2 domain to the IgG domain, or physical conditions during fusion protein preparation that may be incompatible with preservation of enzyme activity. (Fig. 15) shows the relative potency of Fc-silencing technologies, determined by SPR measurements of the binding of purified, recombinant protein samples of each modified antibody to immobilized recombinant human FcγRI receptor. At the far right, both Paradigm’s Fc-silencing method and mAbsolve’s STR silencing methods show the lowest, almost undetectable binding to FcγRI receptor. The Inset displays SPR data for binding of either the LiVE (non-YTE) or LiVE-Longer chimeras to purified FcRn receptor; note high affinity binding of the YTE chimera to FcRn, but not by the non-YTE variant; binding to FcRn will increase the biological half-life of the chimera in nasal epithelium (see below and Discussion). (Fig. 16) shows data underlying the inclusion of the YTE mutations in the “LiVE-longer” version of the ACE-2 LVE/STR chimera. Reprinted here with publisher’s permission, Ladel *et al*. [24] showed that IgG binding to the FcRn receptor, which is permitted by the YTE mutation, results in slow (4–8 h) transcytosis and recycling of the IgG in the nasal mucosa, which results in a much longer biological half-life of IgGs administered nasally.

 (Table 1) is a summary of the measured binding affinities, determined by SPR assay, of Paradigm’s LiVE and LiVE-longer chimeric constructs to purified recombinant RBD subunits, S1 subunits containing the RBD, or S1 subunit trimers designed to mimic the Alpha, Delta or Omicron variants of SARS-CoV-2. For comparison, the data are displayed alongside binding data for w.t. ACE-2/Fc fusion proteins from ACRO Biosystems and Genscript, which have binding affinities of 27, 16 and ~ 3 nM, respectively, to the Omicron BA4.6 spike protein trimer and Wuhan strain S1 subunits, respectively. In contrast, all Paradigm constructs had low-to-mid picomolar binding affinities to the Alpha, Delta or Omicron variant protein constructs, all orders of magnitude higher affinity than the commercial w.t. hACE-2 constructs. Of special note, the highest affinity bindings were observed for the YTE variant “LiVE Longer” chimera to the Omicron subvariant BA.2 spike trimer (78 fM), to the Omicron subvariant BA2.75 spike trimer (133 fM), to the Omicron subvariant spike trimers BA.1 (73 pM), BQ.1.1 (1.81 pM) and to the Alpha B.1.1.7 variant (93 pM). These data are consistent with the sVNT assays described above; implications for potential therapeutics are described in the Discussion section.

## DISCUSSION

This manuscript describes a new generation of chimeric ACE-2/Fusion protein hybrid molecules designed to have four important characteristics: (a) ultra-high affinity binding to viral targets; (b) preservation of high affinity binding across variant subgroups; (c) the option of strong silencing of Fc receptor function to minimize ADE of infection or complement antibody-dependent enhancement (C’ADE) and (d) the option of binding to the FcRn receptor to increase biological half-life, particularly in upper respiratory passages. Given the rapid and consistent evolution of new variants of SARS-CoV-2 in the past 2 years, it is proposed that this class of chimeric molecules offers a viable new set of approaches to prophylaxis of SARS-CoV-2 and, in the future with alternate designs, other emerging viral threats yet to come.

An important feature of the design presented here is the choice of mutations to engineer into the viral receptor portion of the chimera, which for SARS-CoV-2 is the human ACE-2 protein. Multiple modeling platforms were compared and were used to choose the “L-V-E” ACE-2 variant described; at each step, the modeling results were compared favorably to recently published crystal and cryo-EM structures of ACE-2 bound to SARS-CoV-2 variants. The substitution of E90 for the w.t. N eliminates the N-linked glycan, which relieves steric hindrance by the sugar and allows closer ACE-2/RBD interactions with all other mutants tested (see Fig. 3). The AA substitutions L27 and V34, which interact with SARS-2 RBD AAs 473 and 456 versus 455 and 453, respectively (Fig. 2), were found in modeling to produce the most stabilizing ACE-2/RBD interactions (lowest D-FIRE score and K_D_ by SPR) for the widest number of SARS-2 variants, especially when paired with the E90 substitution to eliminate the N-glycosylation (See Figs 4 and 5 and Table 1). Somewhat surprisingly, when the LVE mutant of ACE-2 was paired with the YTE sequence in the IgG portion of the chimera, the measured binding affinities to several SARS-2 variants were even greater than those measured in the non-YTE construct (to be discussed below, see Table 1). Moreover, the sVNT and infection assays reported in (Figs 7–9) and 11–13 yielded viral neutralization data entirely consistent with the modeling and SPR binding data.

Of particular note in the context of the most recent SARS-CoV-2 Omicron sequence at the time of manuscript submission, the SARS CoV-2 RBD mutation N417 (w.t. is K417), along with other Omicron mutations viewed in 3D molecular modeling (see Fig. 10, top panel), has caused this RBD AA to move further away from the ACE2 D30 AA and adopt a more vertical orientation (purple arrow next to V34), compared with the w.t. K417 which was more horizontal (not shown). Across multiple modeling simulations, the choice of valine at ACE-2 position 34 offered the widest variety of favorable ACE-2/RBD interactions, including with the recent BA.1, BA.2 and BA.5 sublineages of Omicron which have lost the K417 mutation. It is for these reasons that the chimeric molecules described here may be termed “variant agnostic”. Of note, substitution of a tyrosine for the leucine at position 27 (Fig. 13, far right “Y-V-E”) resulted in reduced inhibition of viral replication by Omicron subvariant BA.5, when compared with the otherwise identical LVE fusion protein, possibly due to the greater size of the tyrosine side chain relative to leucine. Interestingly, a very recent report describing the relatively new VOC BA.4.6 showed that although BA.4.6 has mutations that allowed nearly complete escape from neutralizing antibodies such as Evusheld, the mutation R346 did not affect binding to ACE-2 [28]. For these reasons, it was expected and demonstrated that BA.4.6 bound the fusion proteins described here with very high affinity (845 pM, see Table 1).

As suggested in (Fig. 6), measured binding affinities determined with S1 protein mimics (top panel) versus the RBD only (bottom panel) may have slower off-rate (longer plateau phase). This might possibly be due to the additional residues in the S1 subunit compared with the RBD alone, through some uncharacterized interaction(s) between those additional residues and either the ACE-2 or IgG subdomains of the fusion proteins. In this particular case, the S subunit versus RBD of two different VOCs were analyzed (Alpha vs. Delta variants in Fig. 6, respectively), so it is unclear if the difference in off-rate was due to variant sequence or the size of the mimic analyzed.

The intentional inclusion of the YTE variant of the antibody domain of the chimera was designed to permit increased binding of the “LiVE-Longer” chimeras to the FcRn receptor, which is known to increase the biological half-life of other IgGs currently in use by 3- to 4-fold [25, 29]. The FcRn receptor binds primarily to the CH2/CH3 interdomain area on IgG Fc, but the Fab arms also contribute to FcRn binding. For this reason, some fusion proteins such as TNFR-IgG Fc mABs (etanercept, trade name Enbrel) have a substantially shorter half-life than normal IgG [30]. For this reason, the incorporation of the YTE sequence in Paradigm’s chimeras is expected to not only saturate the FcRn widely expressed in the respiratory tract, but is predicted to substantially increase their biological half-life. In further support of this prediction, Motavizumab-YTE and Omalizumab-YTE have both been shown to have an extended half-life in healthy adults simply as a result of incorporating the YTE sequence [31, 32], a property known to be imparted by binding of this sequence to the FcRn receptor [33]. Although biological half-life has not yet been tested for the fusion proteins described here, future pharmacokinetic and pharmacodynamic studies to be performed as part of a later FDA EUA submission are expected to yield a similar half-life extension of 2–4-fold.

This is a feature that no other ACE2-Fc fusion proteins to date have taken into account, and is expected to allow lower doses, administered less frequently, to achieve therapeutic efficacy. By analogy to other mAbs containing the YTE sequence [25], it is expected that the Paradigm chimeras expressing YTE will exhibit 3–4-fold increased biological half-life, especially if administered nasally, due to high FcRn expression in the nasal and oral epithelia [16]. The SPR data of (Fig. 15) (see inset) are consistent with this hypothesis, as the YTE construct exhibited nearly 20-fold higher binding affinity to purified FcRn (27 nM) compared with the non-YTE construct (517 nM). The lower pH of the nasal cavity (~5.5, [34]) is not expected to decrease ACE-2 binding, as computational modeling of chimera-RBD binding at pH 7.4 versus 5.5 yielded DFIRE Scores of −8.54 versus −8.01, respectively (data not shown). In addition, processing of the LiVE Longer fusion protein through a commonly available home-use nebulizer had no significant effect on the ability of the chimera to neutralize Omicron variants in the sVNT assay (data not shown), supporting the proposed delivery of the fusion proteins by inhalation/nasally.

In addition, and somewhat surprisingly, the LiVE-Longer YTE chimeras, when compared with their non-YTE counterparts, also showed consistently higher binding affinities to the SARS-2 protein constructs corresponding to the Alpha variant B.1.1.7 and the Omicron variants B.1.1.529 and BA.1, when these were assayed as S1 subunits or S1 subunit trimers (see Table 1). Furthermore, the highest affinity binding was found for the YTE chimera to the Omicron subvariant BA.2 (78 fM). The reason for this increased viral binding by the YTE variant is not presently clear, but may be related to the YTE variant increasing IgG hexamer formation (personal communication, Dr Neil Bodie). Another potential benefit of incorporating the YTE sequence for FcRn binding is the presence of FcRn expressed by endothelial cells throughout the vasculature [35]. Recently, extracellular vimentin expressed and released by endothelial cells was shown to act as an adjuvant to ACE-2, increasing ACE-2-mediated entry of SARS-CoV-2 into the endothelium and thereby promoting infection [36]. In light of these findings, it might be predicted that high binding of the YTE chimeras to FcRn within the vasculature, together with the increased half-life that binding imparts, would act to further inhibit vimentin-mediated ACE-2-dependent cell entry by the virus. Whether or not these hypotheses are correct will be interesting topics for future investigations.

Regardless, the new chimeric ACE-2/Fc-silent fusion proteins described here offer a promising new approach to prophylaxis of SARS-CoV-2 infection that rigorous pre-clinical testing has shown to be relatively variant-agnostic. On the basis of published data from other mAb preparations containing the YTE sequence, the biological half-life of these constructs is expected to be increased 3–4-fold above that of non-YTE fusion proteins. This feature is expected to not only increase biological half-life, but due to the high expression of FcRn in nasal and oral mucosa, enable lower and less frequent dosing of compound delivered intranasally. Given the stability of these constructs at the acidic pH of the nasal mucosa, it is proposed that intranasal delivery or nebulization may be an optimal delivery route for this proposed prophylactic strategy against SARS-CoV-2 infection. By saturating the respiratory tract FcRn with the “LiVE Longer” mAb, the goal of these YTE prophylactics is to achieve passive sterilizing immunity in future in vivo pre-clinical and human clinical testing. In addition, the design described here offers the possibility to exchange the ACE-2 portion of the construct with other viral receptors, in future efforts to combat viral threats that are likely to emerge.

## CONCLUSIONS

In this report, we describe the design of new molecules which combine a synthetic human ACE-2 domain that is mutated to allow variant-agnostic, ultra-high affinity binding to the SARS-CoV-2 S1 subunit. The ACE-2 domain is combined with an Fc-silent antibody domain that essentially eliminates the potential for ADI or ADE. Moreover, a third mutant option of the antibody domain of the chimera is offered, with the intent to substantially increase (3–4-fold) the biological half-life of the chimera, especially if delivered by aerosol or nasal administration. It is proposed that a nasal administration of the new chimeric molecules described herein will constitute an effective prophylactic against SARS-CoV-2 infection that will not only be effective, but also will be economically superior to current monoclonal antibody treatments for COVID-19.

## Data Availability

The authors confirm that the data supporting the findings of this study are available within the article [and/or] its supplementary materials.

## ANIMAL RESEARCH STATEMENT

No animal studies were conducted in this research.
